# Comprehensive review of the evidence regarding the effectiveness of community–based primary health care in improving maternal, neonatal and child health: 2. maternal health findings

**DOI:** 10.7189/jogh.07.010902

**Published:** 2017-06

**Authors:** Mary Carol Jennings, Subarna Pradhan, Meike Schleiff, Emma Sacks, Paul A Freeman, Sundeep Gupta, Bahie M Rassekh, Henry B Perry

**Affiliations:** 1 Department of International Health, Johns Hopkins Bloomberg School of Public Health, Baltimore, MD, USA; 2 Institute for Global Health, Duke University, Durham, North Carolina, USA; 3 Independent Consultant, Seattle, Washington, USA; 4 Department of Global Health, University of Washington, Seattle, Washington, USA; 5 Medical Epidemiologist, Lusaka, Zambia; 6 The World Bank, Washington, District of Columbia, USA

## Abstract

**Background:**

We summarize the findings of assessments of projects, programs, and research studies (collectively referred to as projects) included in a larger review of the effectiveness of community–based primary health care (CBPHC) in improving maternal, neonatal and child health (MNCH). Findings on neonatal and child health are reported elsewhere in this series.

**Methods:**

We searched PUBMED and other databases through December 2015, and included assessments that underwent data extraction. Data were analyzed to identify themes in interventions implemented, health outcomes, and strategies used in implementation.

**Results:**

152 assessments met inclusion criteria. The majority of assessments were set in rural communities. 72% of assessments included 1–10 specific interventions aimed at improving maternal health. A total of 1298 discrete interventions were assessed. Outcome measures were grouped into five main categories: maternal mortality (19% of assessments); maternal morbidity (21%); antenatal care attendance (50%); attended delivery (66%) and facility delivery (69%), with many assessments reporting results on multiple indicators. 15 assessments reported maternal mortality as a primary outcome, and of the seven that performed statistical testing, six reported significant decreases. Seven assessments measured changes in maternal morbidity: postpartum hemorrhage, malaria or eclampsia. Of those, six reported significant decreases and one did not find a significant effect. Assessments of community–based interventions on antenatal care attendance, attended delivery and facility–based deliveries all showed a positive impact. The community–based strategies used to achieve these results often involved community collaboration, home visits, formation of participatory women’s groups, and provision of services by outreach teams from peripheral health facilities.

**Conclusions:**

This comprehensive and systematic review provides evidence of the effectiveness of CBPHC in improving key indicators of maternal morbidity and mortality. Most projects combined community– and facility–based approaches, emphasizing potential added benefits from such holistic approaches. Community–based interventions will be an important component of a comprehensive approach to accelerate improvements in maternal health and to end preventable maternal deaths by 2030.

Traditionally, maternal health programs in low–income settings have focused on improving the access to and quality of clinical services provided in health facilities. However, increasing facility delivery alone is likely insufficient for further substantial reductions in maternal mortality and morbidity [1,2]. The contribution that community–based primary health care (CBPHC) can make to improving maternal health has received much less attention. Although ready access to and appropriate utilization of primary health care centers and referral hospitals is essential to manage pregnancy complications [3,4], an increasing number of community–based interventions have been designed in an effort to accelerate improvements in maternal health.

Although improving maternal health by increasing the access to and the quality of maternal health care services has been acknowledged as a global health priority, recent progress in improving maternal health in low–income countries has been discouragingly slow, particularly in sub–Saharan Africa and parts of South Asia [5]. The Millennium Development Goal 5 (reducing maternal mortality by 75% between 1990 and 2015) was not met: only a 44% decline has been achieved globally – representing a decline from 385 to 216 maternal deaths per 100 000 live births between 1990 and 2015 [6].

The purpose of this paper is to review the available evidence regarding the effectiveness of CBPHC in improving maternal health broadly defined. It extends the focus of a previous review by Kidney et al. [7] that was limited to controlled studies of the effectiveness of community–level interventions in reducing maternal mortality. It also extends the findings of a recently published review by Lassi et al. (2016) [8] by providing a broader and more in–depth review of community–based approaches to improving maternal health.

This review is derived from assessments of projects, programs and research studies (hereafter referred to as projects) that implemented community–based interventions and measured their impact on maternal health. Our paper is part of a series on the effectiveness of CBPHC in improving maternal, neonatal and child health also reported in this journal [9–14].

## METHODS

We conducted a search on PUBMED for assessments of CBPHC on maternal health. We defined such assessments of effectiveness broadly, as any document that assessed the effect of a CBPHC intervention on maternal health irrespective of inclusion of assessment of outcome on fetal, newborn or child health outcomes. The shared review methods for this series are described elsewhere in this series [9]. In addition, our maternal review searched additional databases including POPLINE, the Cochrane Review system, and CABI Publishing Database Subsets to identify additional documents. We included assessments identified from review articles. We made requests to knowledgeable professionals and organizations in the field of global public health for further listings of documents to be considered for inclusion. In order to provide a comprehensive set of documents that not only included clinical trials but also quasi–experimental designs, pre–post comparisons, program evaluations, and general descriptions of intervention effect, we used broad inclusion criteria.

Documents were eligible for inclusion in the present assessment if they: (1) involved an intervention intended to improve maternal health; (2) included interventions that took place outside of a health facility; (3) measured a change in maternal health (mortality, morbidity, nutritional status, or population coverage of a key maternal service) (eg, antenatal care attendance, facility–based delivery, attended delivery); (4) assessed an activity targeting a change in maternal health. We defined CBPHC as a health intervention with a community component based outside of a physical health facility.

Two of the authors (HP, MJ) reviewed the abstracts of 7890 articles published on PUBMED through December 2015. Of these, 120 met criteria for inclusion. Additionally, 33 documents that were identified from the gray literature through searches of personal and colleague databases met criteria for inclusion. A total of 152 assessments met the final inclusion criteria. Two reviewers independently abstracted information from these assessments using a standardized data extraction form; a third independent reviewer resolved any discrepancies between the initial two reviews to provide a final summative review. The data were transferred to an electronic database and initially analyzed in EPI INFO version 3.5.4 (Epi Info, US Centers for Disease Control and Prevention, Atlanta, Georgia, USA). Microsoft Excel (Microsoft, Seattle WA, USA) was used for additional descriptive analyses. Appendix S1 in **Online Supplementary Document(Online Supplementary Document)** contains the references for these 152 assessments; the assessments and year cited in the main text in parentheses are followed by the letter “S” and a number indicating the order of the reference in Appendix S1 in **Online Supplementary Document(Online Supplementary Document)**. In the tables, these assessments are cited by the first author and year followed in parentheses by the letter “S” and a number indicating the order of the reference in Appendix S1 in **Online Supplementary Document(Online Supplementary Document)**.

Reviewers who extracted data defined outcome indicators as primary and secondary depending on the type of project and its goals. In general, primary outcomes had study designs that provided sufficient power to detect a statistically significant difference in that outcome, while assessments of secondary outcomes were not similarly powered. Here we describe the basic characteristics of the full database of maternal articles and present a more detailed descriptive analysis of documents from this database that measured the effects of interventions on the primary outcomes of maternal mortality and morbidity. We describe the key characteristics of the interventions employed by each project as well as the strength of evidence of effectiveness. We include descriptions of documents that failed to report significance or reported statistically insignificant effects to provide a fair representation of the field and to avoid only reporting positive results.

To more fully explore the impact of community–based interventions on maternal health outcomes, we make a brief description of changes in the population coverage of antenatal care, attended delivery, and facility–based delivery. However, including these in as detailed an assessment as we have conducted for primary mortality and morbidity outcomes will be reserved for a subsequent article.

## RESULTS

### Community settings

Bangladesh, India, Pakistan and Nepal were the location of the largest number of assessments (16, 15, 14 and 11, respectively). Data from a total of 169 countries were included in these 152 assessments. Six assessments included data from multiple countries in multiple regions. Countries were from six geographic regions, with the majority of them in South–East Asia (41%) and West Sub–Saharan Africa (22%). The majority of the 152 assessments were performed in rural communities (83%), with 11% in peri–urban and 10% in urban locations. The largest percentage (48%) of the 152 assessments were performed for an intervention that took place at the district or sub–province level; 8% took place at the province level; and 3% at the national level. 30% of interventions took place in a group of communities, and 9% took place in a single community.

### Interventions

**Figure 1:**
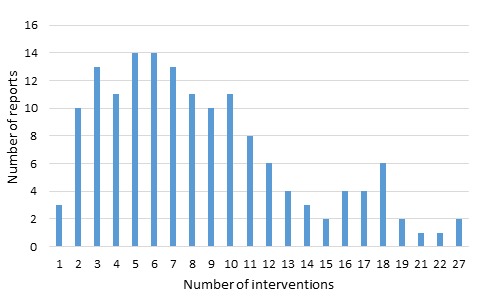
Number of interventions implemented in individual assessments of the effectiveness of community-based primary health care in improving maternal health.

Each assessment described the effectiveness of one or more discrete interventions, ranging in number from 1 to 27. (A copy of the data extraction form is contained in Online Supplementary Document of another paper in this series [9]). As shown in **Figure 1**, a small number of assessments (2%) described the implementation of only one intervention; a majority (72%) of the documents described packages comprised of between 1 and 10 interventions.

In total, the 152 assessments described 1298 discrete interventions. 57% of these interventions promoted or provided routine maternal health care. These activities included antenatal and postpartum visits, immunizations, attendance of a skilled attendant at delivery, or making referrals to higher levels of care. 37% of these interventions addressed medical complications of pregnancy. These activities included screening and treating medical conditions such as high–risk pregnancy, gestational diabetes, hypertensive disorders, and infections. 6% of these interventions targeted socio–economic conditions of the mother, including participation in micro–credit and savings groups, conditional cash transfers, women’s empowerment programs, and participatory women’s groups.

**Box 1** describes three examples of intervention packages from three assessments with a larger number of kinds of interventions.

Box 1 **Examples of community–based intervention packages for improving maternal health**

**Example 1.** A community–based package implemented in 12 villages in rural India included the following interventions [15]:

- Provision of services at outreach sites by facility–based providers
- Provision of weekly antenatal clinics at outreach sites
- Provision of home visits for antenatal care by public health nurses
- Provision of treatment for simple illnesses by community health workers
- Provision of maternal education on child birth, child care, breastfeeding, immunizations, family spacing, and home economics by community health workers
- Distribution of iron/folate tablets in the community
- Identification of high–risk mothers in the community by community health workers and referral to a higher level of care

**Example 2.** A community–based package implemented in eight states in northern India included the following interventions [16]:

- Provision of antenatal and postnatal home visits by health workers
- Provision of tetanus immunizations
- Provision/promotion of iron–folic acid tablets
- Behavior change messages to promote saving money for birth planning and childbirth;
- Promotion of delivery at a facility and, if a home delivery is planned, promotion of the use of a skilled birth attendant
- Promotion of immediate postpartum breastfeeding

**Example 3.** A package of community–based interventions implemented in four districts of rural Bangladesh [17]:

- Formation of village health committees
- Training and linking traditional birth attendants to community health workers
- Promotion of family planning
- Identification of pregnancies at an early stage
- Promotion of birth planning
- Promotion of delivery by a trained health worker
- Promotion of immediate and exclusive breastfeeding
- Provision of antenatal care, delivery care, and postnatal care
- Promotion of vaccinations for pregnant women
- Referral for maternal complications
- Facilitate access to clinical services in health facilities

### Categorization of outcome indicators

The 152 assessments described a multitude of outcome indicators. Categorization of outcome indicators aids in the assessment of intervention effectiveness. We extracted counts of indicators in five categories: (1) maternal mortality, (2) maternal morbidity, (3) population coverage of antenatal care attendance, (4) population coverage of deliveries by a skilled provider or a trained traditional birth attendant, and (5) population coverage of deliveries taking place at a facility. 19% of the assessments included maternal mortality as an indicator, and 21% measured maternal morbidity. In addition, 50% of the assessments measured antenatal care attendance; 66% measured attendance of deliveries by a skilled provider or trained traditional birth attendant; and 69% measured facility deliveries. A complete list of the outcome indicators among these 152 assessments is included in Online Supplementary Document of another article in this series [9].

### Impact on maternal mortality

A maternal death was defined in the majority of assessments according to WHO definition: the death of a pregnant woman or a woman within 6 weeks of cessation of pregnancy, from any cause related to the pregnancy or its management, but excluding accidental causes. Of the 32 documents that assessed maternal mortality, 15 assessed mortality as a primary outcome indicator (**Table 1**). Of the 7 assessments that performed statistical significance testing, 6 reported significant decreases in mortality ranging from 42% to 78% and 1 suggested a trend toward increased mortality but this effect was not significant (**Table 1**). This suggestion of an increased mortality effect was only present when the facility–based intervention was analyzed together with the community arm, in comparison to the control arm. The suggestion of effect reversed in direction when the community arm was considered by itself against the control, with a 9% (non–significant) reduction in odds of maternal mortality rate (odds ratio OR 0.91, 95% confidence interval CI 0.51–1.63) (Colbourn 2013, reference [S39] in Appendix 1 in **Online Supplementary Document(Online Supplementary Document)**). The design of the remaining 8 assessments with maternal mortality as a primary outcome did not permit statistical testing, but in all these assessments there was suggestion of decrease in maternal mortality. These reports suggested substantial impact, with four reporting a reduction to zero maternal deaths post–intervention (Asha–India 2008 [S19]; Curamericas Guatemala A&B 2007 [S41–42]; Lamb 1984 [S73]; Emond 2002 [S47]) and the remainder suggesting substantial decreases compared to regional or national population–level comparisons.

**Table 1:** Effect size, direction and significance of community–based primary health care on maternal mortality outcomes*

Document	Intervention type	Effect	Study population	Effect size and confidence interval	Significance level†
Colbourn 2013 [S39]	Participatory women's groups in the community and quality improvement at health centers	Increase	Two–by–two factorial cluster randomized controlled trial of community compared to facility intervention, 14 576 births during baseline and 20 576 births during intervention, in 3 districts in rural Malawi, over 29 months from 2007–2010	8% increase in odds of maternal mortality in facility + community arm compared to control (OR: 1.08, 95% CI, 0.46–2.57)	*P* = 0.854
Manandhar 2004 [S83]	Participatory women's groups in the community, with 9 meetings per month and action–learning cycle	Decrease	Cluster–randomized controlled trial of 12 pairs of community clusters in 28 931 women in rural Nepal, over 2 years from 2001–2003	78% decrease in odds of maternal mortality in community intervention clusters compared to control clusters (OR: 0.22, 95% CI, 0.05–0.90), a maternal mortality ratio of 69 compared to 341 per 100 000 live births, respectively	**Significant, based on confidence interval** (*P* value not reported)
Zhenxuan 1995 [S152]	Linked community–based mass health education campaign with facility– and community–based strengthening of emergency services	Decrease	Quasi–experimental pilot study compared to control area, covering 8000 deliveries per year in one county in peri–urban China, over 3 years from 1985–1988	Maternal mortality ratio (per 100 000 live births) decreased by 75.7% in the intervention areas and by 5.5% (*P* > 0.05) in the control areas	***P* < 0.001**
Seim 2014 [S128]	Community mobilization to identify and refer protracted labor cases	Decrease	Pilot impact assessment, 12 254 births in rural Niger over 3 years from 2008–2011	Birth–related maternal mortality ratio fell by 73% over 3 y, from 630 to 170 per 100 000 live births	***P* < 0.001**
Koenig 1988 [S70]	Provision of community–based family planning services	Decrease	Quasi–experimental study compared intervention to control areas using demographic surveillance data from 187 523 people in 149 villages, 70 in intervention and 79 in control, in Matlab, Bangladesh over 9 years from 1976–1985	Significant overall decrease in maternal mortality rate for intervention vs control (66 vs 121 deaths per 100 000 women of childbearing age) but no significant change in maternal mortality ratio (effect size not reported)	***P* < 0.001**
Fauveau 1991 [S51]	Provision of antenatal and maternity care and surveillance of vital events in the home and community	Decrease	Non–randomized evaluation of intervention villages compared to neighboring non–intervention villages with 196.000 total population, in rural Bangladesh over 3 years from 1978–1981	65% decrease in odds of maternal mortality in intervention compared to control area (OR: 0.35, 95% CI, 0.13–0.93), or 140 vs 380 per 100 000 live births	***P* < 0.05**
Fauveau 1990 [S50]	Provision of primary and preventive care (maternal and child) in the home and community	Decrease	Non–randomized evaluation of intervention villages compared to neighboring non–intervention villages with 196 000 total population, in rural Bangladesh over 3 years from 1978–1981	42% lower rate of maternal mortality in control vs intervention (authors reported RR in control over intervention: RR 1.73, 95% CI, 1.02–2.93) (rate of 5.0 vs rate of 8.6 per 10 000 women of child–bearing age)	***P* < 0.05**
Asha–India 2008 [S19]	Provision of community–based primary and antenatal care and women's empowerment in slum communities	Decrease	Program evaluation of intervention population of 300 000 people in urban slums in India, over 20 years, reporting data from 2007–2008	Zero deaths in Asha slums compared to 540 per 100 000 live births in India country–wide	N/A (maternal mortality ratio in slum areas compared to overall country ratio)
CARE Nicaragua 2008 [S33]	Increase access and improve quality of maternal services through linking communities to facilities and through community mobilization and communication campaign	Decrease	Program evaluation of intervention in population of 174 367 (58 052 women of reproductive age) in 173 rural communities in Nicaragua over 5 years from 2002–2007	Maternal mortality rate decreased from 150 to 34 per 100 000 live births, with an annual average of 5500 deliveries over the 6 years of the intervention; maternal mortality ratio for the entire intervention area decreased from 119 to 60 per 100 000 live births over that time as well (a decrease of 49.2% compared to a national decrease of 42.6%)	N/A (maternal mortality rate decreased from baseline to endline in the primary referral hospital intervention area)
Curamericas–Guatemala–A&B 2007 [S41–42]	Care Groups and community–based impact–oriented care delivery/surveillance	Decrease	Program evaluation of intervention in population ranging in size from 11 123 (at end evaluation) to 14 272 (at mid–point) women of reproductive age, in 3 rural municipalities in Guatemala over 5 years from 2002–2007	Maternal mortality ratio decreased in all intervention areas relative to national data used as control (508 per 100 000 live births to zero, and 1124 per 100 000 live births to zero, over 4 years of data)	N/A (not powered sufficiently for statistical testing; diverse results)
Foord 1995 [S54]	Provision of primary and antenatal care in the community, and establishment of referral linkages	Decrease	Non–randomized evaluation of intervention compared to similar control area, each with a population of 1300, in a rural district of the Gambia over 2 years from 1989–1991	1 death in intervention area compared to 5 deaths in control area, giving a maternal mortality ratio of 130 per 100 000 live births in the intervention compared to 700 in control area	N/A (not powered sufficiently for statistical testing)
Lamb 1984 [S73]	Provision of direct medical care, nutrition and vital statistics surveillance in community	Decrease	Non–randomized non–controlled evaluation of intervention impact in 4 villages with total population of 2000, in rural Gambia over 10 years from 1974–1984	No pregnancy–related deaths (per 1000 women of child bearing age) were observed in the community for the 8 years of intervention, compared to the annual 16 that would be expected using rates in comparable non–intervention areas	N/A (not powered sufficiently for statistical testing)
Emond 2002 [S47]	Provision of antenatal care in the community	Decrease	Non–randomized non–controlled evaluation of an intervention in a population of 42 000 in an urban district in Brazil over 30 months from 1995–1997	Maternal mortality ratio decreased from 335 per 100 000 live births prior to intervention, to zero maternal deaths during the 1 year after the intervention	N/A (not powered sufficiently for statistical testing)
Purdin 2009 [S117]	Community education campaign and creation of emergency obstetric centers linked to primary care centers	Decrease	Non–randomized non–controlled evaluation of intervention among community of 96 300 Afghan refugees in Pakistan over 4 years from 2004–2007	Annual maternal mortality ratio decreased from 291 to 102 per 100 000 live births over 4 years	N/A (baseline and endline rates calculated from two separate sources)
Findley 2015 [S53]	Behavior change and health systems integration	Decrease	Non–randomized evaluation of intervention compared to control and before compared to after, of 2360 women at baseline and 4628 at follow–up, in 3 states in northern Nigeria over 4 years from 2009–2013	Estimated maternal mortality ratio showed a larger decrease in the intervention than in the control communities, from 1270 to 1057 (interventions) and to 1262 (controls) per 100 000 live births	N/A (based on estimates)

N/A – not available; RR – rate ratio, CI – confidence interval, OR – odds ratio

* For assessments in which maternal mortality was the primary outcome indicator. The full references are shown in Appendix S1 in **Online Supplementary Document(Online Supplementary Document)**.

† Significant results indicated in bold font.

### Impact on maternal morbidity

29 of the 152 assessments measured changes in maternal morbidity, most commonly measuring postpartum hemorrhage (14 assessments), anemia (13), eclampsia (8) or malaria (6). Of these 29 documents that assessed maternal morbidity, 7 assessed a discrete morbidity as a primary outcome indicator and so are described in **Table 2**. Six of these assessments reported a significant decrease in at least one of the maternal morbidity indicators; one assessment suggested a decrease but did not report significance testing, and none reported a worsening of maternal morbidity.

**Table 2:** Effect size, direction and significance of community–based primary health care on maternal morbidity outcomes*

Reference	Intervention type		Effect	Population	Effect size and confidence interval	Significance level†
**Incidence of postpartum hemorrhage (PPH)**						**PPH, Severe PPH‡**
Derman 2006 [S45]	Auxiliary nurse midwives (ANMs) administered oral misoprostol (or placebo) at home births they attended	Decrease		A randomized placebo–controlled trial assigned 812 women to oral misoprostol and 808 to placebo after home–based delivery by 25 ANMs, in rural India over 3 years from 2002–2005	47% decrease in incidence of PPH (6.4% in intervention vs 12.6% in control, RR: 0.53, 95% CI: 0.39–0.74); 83% decrease in severe PPH (0.2% in intervention vs 1.2% in control, RR: 0.16, 95% CI: 0.04–0.91). 1 case PPH prevented for every 18 women given chemoprophylaxis	**PPH *P* < 0.001, severe PPH *P* < 0.001**
Mobeen 2011 [S95]	Trained traditional birth attendants (TBAs) administered misoprostol (or placebo) at home deliveries they attended	Decrease		A randomized double–blind placebo-controlled trial assigned 534 women to oral misoprostol and 585 to placebo after home–based delivery by 81 TBAs, in one province in rural Pakistan over 24 months from 2006–2007	24% reduction in PPH after delivery (16.5% in intervention vs 21.9% in control, RR: 0.76, 95% CI 0.59–0.97); Insignificant decrease in severe PPH (RR: 0.57, 95% CI: 0.27–1.22)	**PPH *P* < 0.05;** NS
Stanton 2013 [S138]	Community health officers injected prophylactic oxytocin (or placebo) at home births they attended	Decrease		A community–based, cluster–randomized controlled trial assigned births conducted by 54 community health officers were randomized to study arm by officer, in 4 rural districts in Ghana, 689 in intervention and 897 in control, over 19 months from 2011–2012	Reduction of 51% in PPH (2.6% in intervention vs 5.5% in control, RR: 0.49, 95% CI: 0.27–0.88) No significant change in severe PPH (1 case in intervention, 8 in control group)	**PPH *P* < 0.05**; NS
**Prevalence of malaria and anemia in malaria treatment interventions**						
Mbonye 2008–5 [S90]	4 cadres of community health workers administered intermittent preventive treatment (IPT) for malaria in pregnancy in the community, compared to routine care in health clinics	Decrease		A non–randomized community trial assigned 2081 women (21 communities) to intervention and 704 women (4 communities) to control in 9 sub–counties of one district in central, rural Uganda over 21 months from 2003–2005	Prevalence of malaria episodes decreased from 49.5% to 17.6% in intervention and from 39.1% to 13.1% in control (both *P* < 0.001). 67.5% of women in the community–based intervention received IPT compared to 39.9% in facility–based control (*P* < 0.001)	***P* < 0.001;** Significance for RR difference in reported malaria was not reported
Mbonye 2008–3 [S89]	4 cadres of community health workers administered intermittent preventive treatment for malaria in pregnancy in the community, compared to in health clinics	Decrease		A non–randomized community trial assigned 2081 women (21 communities) to intervention and 704 women (4 communities) to control in 9 sub–counties of one district in central, rural Uganda over 21 months from 2003–2005	Decreased prevalence of reported malaria episodes in both community and facility distribution of IPT (64% in community, from 49.5% to 17.6%, vs 66% decrease in facilities, from 39.1%, to 13.1%) (both *P* < 0.001)	***P* < 0.001** [Significance for RR difference in reported malaria was not reported)
Ndiaye 2009 [S105]	Positive deviance program using community–based volunteers to promote maternal health and nutrition, and to distribute iron supplements, to control anemia during pregnancy	Decrease (improvement)		A quasi–experimental design using pre–post evaluation of independent cross–section samples assessed 371 women in one community in rural Senegal over 9 months in 2003	75% reduction in risk of anemia, based on mean hemoglobin measurements, in the intervention compared to control area (no positive deviance) (OR: 0.25, 95% CI: 0.12–0.53)	***P* < 0.003§**
**Eclampsia**						
Shamsuddin 2005 [S130]	Quasi–experimental study involving community, home–based administration of magnesium sulfate to diagnosed eclamptic and severe eclamptic cases prior to referral to hospital, compared to control cases who did not receive injections	Decrease		256 cases from 3 districts in Bangladesh, 133 in intervention and 132 in control, over 6 months in 2001	Decreased number of mean convulsions in the intervention cases (4.7 ± SD2.64) compared to control cases (6.86 ± SD 2.97) (*P* < 0.001)	***P* < 0.001**

CI – confidence interval, SD – standard deviation, OR – odds ratio, PPH – postpartum hemorrhage, NS – not (statistically) significant, RR – rate ratio

*For assessments that analyzed maternal morbidity as a primary outcome indicator. The full references are shown in Appendix S1 in **Online Supplementary Document(Online Supplementary Document)**.

†Significant results indicated in bold font.

‡PPH defined in each assessment as blood loss ≥500 mL; severe PPH defined in each assessment as blood loss ≥1000 mL.

§Chi–square test of difference between control and intervention.

### Postpartum hemorrhage

Three of the seven documents measured change in postpartum hemorrhage following a preventive intervention delivered by a community health worker. These documents used the standard definition of measured blood loss greater than or equal to 500mL, and defined severe postpartum hemorrhage as blood loss greater than or equal to 1000mL (Kapungu 2013 [S65]; Fauveau 1990 [S50]; Derman 2006 [S45]). The three measurements of reduction in postpartum hemorrhage were statistically significant, with decreases ranging from 24% to 66% (**Table 2**). One assessment reported a significant decrease in severe postpartum hemorrhage, and the remaining two did not have a significant effect on severe postpartum hemorrhage.

### Malaria

Two assessments reported measures of primary outcomes related to malaria, including the prevalence of anemia in malaria–endemic areas (two assessments) and the prevalence of maternal malarial episodes (one assessment). Of note, two of these assessments pertained to different aspects of a single intervention but were reported in separate peer–reviewed publications. One document reported equivalent, significant decreases in anemia in both community–based and facility–based intermittent preventive treatment (IPT) of malaria in pregnancy, (mean hemoglobin increased by 6.7% with 2 doses of IPT in both arms) (Mbonye 2008–5 [S90]). However, the women in the community arm received their first dose of IPT as recommended (during the second trimester) more frequently than the women in the facility arm (92.4% in the community vs 76.1% in the facility, *P* < 0.001). Women in the community arm also received IPT at a significantly earlier stage of pregnancy compared to those in the facility arm (21 weeks vs 23 weeks, *P* < 0.001), and the results described significantly higher adherence to the recommended two doses in the community arm compared to the facility arm. The community–based approach increased access to and use of IPT (Mbonye 2008–5, [S90]). The second assessment measured prevalence of reported malaria episodes and reported similar decreases in both community and facility distribution groups, but did not report significance testing of the relative difference in risk (Mbonye 2008–3 [S89]). One report assessed the prevalence of anemia, reporting a significant decrease of 75% in the intervention area vs the control area (Ndiaye 2009 [S105]).

### Eclampsia

One assessment measured frequency of convulsions in eclamptic or pre–eclamptic cases who received magnesium sulfate injections at home prior to hospital transfer, reporting a significant decrease compared to cases who did not receive injections at home (Shamsuddin 2005, [S130]).

### Impact on population coverage of evidence–based interventions

#### Antenatal care

Of the 37 assessments that measured coverage of antenatal attendance as a primary outcome indicator, 34 assessments reported increased attendance for antenatal care (ANC). No assessments observed a decrease in ANC coverage. Three assessments found no change in coverage, and we describe those three here in some detail.

The first assessment that found no change in ANC coverage (Helen Keller International 2003, [S60]) was an evaluation of a pilot program in Mozambique that provided iron and folic acid along with anemia–related health education to communities with a high anemia burden. Both recipient (intervention) and non–recipient (control) barrios showed some increases and some decreases on numerous outcome indicators such as knowledge of anemia, ingestion of iron/folic acid supplements, and reported anemia during most recent pregnancy.

The second assessment with no change in ANC attendance (More 2012 [S97]) was a cluster–randomized controlled trial testing the impact of creating and mobilizing women’s groups in urban slums in Mumbai, India for the purpose of improving perinatal health, including increasing attendance at ANC clinics which had been strengthened through a city–wide maternal and newborn health care program for the urban poor. Although the assessment did report a reduction in the odds of a set of maternal morbidities in the intervention compared to control group (OR 0.60, 95% CI 0.38–0.94), there were no improvements in ANC attendance or other outcomes such as institutional delivery, breastfeeding, care–seeking, stillbirth rate, or neonatal mortality.

The third assessment that found no change in ANC coverage (Langston 2014, [S74]) was a mixed–methods evaluation of integrated community case management for childhood illness that was combined with promotion of maternal ANC attendance. ANC attendance increased in both control and intervention communities, but the difference was not statistically significant.

#### Changes in attended delivery

12 assessments measured coverage of the presence of a skilled or trained attendant at delivery as a primary outcome indicator. All 12 assessments reported an increase in the coverage of attended deliveries. The precise definition of a skilled or trained birth attendant varied among the assessments, and we have not attempted to standardize the definition here. Nine assessments specifically measured percentage of deliveries attended by a “skilled birth attendant,” while one assessment measured the percentage of deliveries attended by a trained traditional birth attendant. Two assessments measured the attendance by a traditional birth attendant as compared to completely unattended deliveries. The two assessments that calculated the statistical significance of coverage changes found a significant increase.

#### Changes in facility–based deliveries

Eight assessments measured the percentage of births occurring in a facility as a primary outcome indicator. None of these assessments observed a decrease in coverage; one observed no change in coverage and seven reported an increase. The types of facilities included in these assessments were hospitals, health centers, and birthing huts.

### Implementers

Community health workers (CHWs) were involved in intervention implementation in 132 of the 152 projects included in our database. In addition to CHWs, project implementers included local government health professionals (78/152 projects), local community members not trained as CHWs (48/152 projects), research staff hired specifically to implement the project (31/152 projects), and expatriates (4/152 projects). Multiple categories of implementers were present in three–fourths (71%) of the individual projects. CHWs were most frequently combined with local government health officials (69 assessments), and with non–CHW members of the local community (40 assessments).

### Implementation strategies

Common strategies used to implement the interventions discussed above are highlighted here.

A typical set of implementation strategies is the following (Baqui 2008 [S24]):

- Used existing government ministry of health infrastructure (facilities and personnel)
- Combined maternal and newborn interventions
- Integrated nutrition with primary care services
- Delivered services and promoted interventions through both skilled and traditional health workers
- Used home visits and health centers to deliver interventions

Community–based strategies used to strengthen maternal health often overlap with community–based strategies to improve neonatal and child health. Strategies to implement community–based interventions for improving neonatal and child health are reported elsewhere [13]. These common strategies include:

- Established community collaborations such as the formation of community health committees
- Engaged community leaders to mobilize communities for a health–related activity
- Formed community groups or collaborated with existing groups (including women’s groups and micro–credit savings groups)
- Engaged communities in the selection and support of CHWs
- Engaged communities in the planning and/or evaluation of CBPHC programming

Home visits were a common strategy used to identify pregnant women, to provide health services and education/counseling, as well as to promote healthy behaviors such as family planning and facility delivery. Home visits were also used to provide postpartum maternal care and identify postpartum mothers with problems requiring referral. The formation and strengthening of participatory women’s groups was a common strategy to motivate women and their families to seek antenatal, delivery and emergency obstetrical care. Outreach visits to the community by a mobile health team based at a peripheral health facility were also a common approach to provide prenatal care, family planning services, and maternal immunizations.

Community–based approaches, particularly through home visits provided by CHWs, are commonly used to increase the coverage of insecticide–treated bed nets for pregnant women and to expand the coverage of intermittent preventive treatment of malaria in malaria–endemic areas. These are interventions that are effective not only for improving maternal outcomes but also for improving perinatal and neonatal outcomes. Community–based approaches to expand the detection of women with HIV infection and to increase the coverage of anti–retroviral treatment of HIV–positive pregnant women include CHWs making home visits and mobile outreach teams.

Health systems strengthening strategies associated with CBPHC for improving maternal health include facilitating referrals (by forming community emergency response committees, community transport systems, and community savings or insurance schemes to cover transport and hospital costs when obstetric emergencies arise). Other health–system–related activities often carried out by projects that also implemented CBPHC interventions included strengthening the quality of care provided at peripheral health facilities (by improving logistics and training staff), and strengthening the supervisory system of community–level workers.

## DISCUSSION

This analysis provides evidence for a positive impact of CBPHC interventions on reducing maternal morbidity, increasing population coverage of evidence–based interventions, and possibly contributing to reductions in maternal mortality in selected settings. Six of the seven assessments that were able to measure the statistical significance of the change in maternal mortality showed a statistically significant decrease. There were eight additional assessments that reported trends in maternal mortality but could not measure the statistical significance of the impact. All eight of these reported a favorable effect on maternal mortality. In contrast to a 2010 Cochrane review of the impact of community–based interventions, which reported reductions in maternal morbidity but no reduction in maternal mortality [18], our inclusion criteria were broad and allowed non–randomized assessments as well as assessments from the gray literature.

All three assessments of the statistical significance of impact of CBPHC interventions on the incidence of postpartum hemorrhage showed significant decreases. One of the three showed a significant decrease in the incidence of *severe* postpartum hemorrhage (which was a secondary outcome for all three projects). Three assessments of CBPHC interventions on maternal malaria and malaria–related anemia all showed significant positive effects, and one assessment of CBPHC interventions on eclampsia showed a significant positive effect.

Our analysis of the effectiveness of CBPHC approaches in increasing the population coverage of evidence–based interventions focused on three interventions: antenatal care attendance, delivery trained provider, and facility–based delivery. Global recommendations for attendance at antenatal care have evolved over time to support increased contacts [19], and the provision of antenatal care as a community–based intervention may help to expand the coverage of more frequent, high–quality and woman–centered pregnancy care in resource–constrained settings.

Delivery attended by a skilled provider improves delivery outcomes [20], but delivery by a fully and formally trained midwife or other highly skilled provider is often beyond the short–term capacity of many countries for all birhts. Strategies that integrate both skilled and traditional birth attendants into the health system are important to increase skilled birth attendance [21,22]. Delivery at a health facility improves access to emergency and critical care for prompt attention to life–threatening maternal complications [3], although the literature points out deficiencies in quality that are commonly observed at facilities [2] and some argue that facility delivery is not a necessary requisite for the reduction of maternal mortality [23,24]. Despite these observations, promoting facility deliveries has been a focus of many interventions aimed at reaching the 2015 Millennium Development Goals for maternal health [25] and now for reaching the 2030 Sustainable Development Goals. However, recent literature suggests that a high rate of institutional delivery by itself is insufficient to reduce maternal mortality ratios [1,26].

A large proportion of the low–income populations globally live more than one hour away from a health facility [4], making utilization of health facilities and emergency care services a challenge. Therefore it is important to strengthen community–based interventions to promote antenatal care attendance, attended delivery, and facility delivery.

The vast majority of community–based primary care interventions described by assessments included in this study were implemented by a wide variety of different types of community–based health workers. It is important to continue efforts to incorporate them in the maternal care process as well as traditional birth attendants, who can serve as doulas (birth companions for facility births) and collaborators in the delivery [27]. Community–based interventions show great potential for reducing morbidity of mothers from malaria and hemorrhage following home delivery.

### Study limitations

Maternal mortality is a rare event, even in settings where maternal mortality is relatively high: even with a maternal mortality ratio of 1000, only 1% of live births are associated with a maternal death. Thus, the demonstration of a statistically significant decline in maternal mortality is a challenge for field programs. As our findings indicate, there are numerous assessments in which there is a suggestion of maternal mortality impact, but the decline does not reach statistical significance. Additionally, there are examples in the literature in which the same community–based intervention shows a statistically significant reduction in maternal mortality in one setting [28] but not in another [29]. One of the explanations for this finding is that the study that did not show a statistically significant change was not adequately powered (meaning that an impact may have been achieved in reality but due to the small sample size it did not reach statistical significance).

This review did not focus on assessments of cost–effectiveness. It is worth noting that studies of the cost–effectiveness of community–based approaches to improving maternal health are rare. Additionally, it is important to note that there are certain settings in which CBPHC may not be effective in improving maternal health – for example in settings where high–quality facility–based care is available and utilized and therefore levels of maternal health are already high. Thus, the cost–effectiveness of CPBHC may be highly dependent on the context. Although evidence of the cost–effectiveness of community–based approaches for improving neonatal and child health care has been summarized [8], there is a need for more research on the cost–effectiveness of community–based maternal health interventions.

The local context in which the assessments were carried out is important to more fully understand which CBPHC components are most useful in which setting. The availability of trained personnel to provide maternity care, the availability and utilization of health facilities, and the local geographic context are all important in assessing how CBPHC can most effectively contribute to improve maternal health. However, to adequately explore these issues is beyond the scope of this paper.

This review did not assess the effects of community–based family planning interventions on maternal health because their effects are indirect and not readily measured in specific program settings, including in the assessments included in our review. However, there is extensive evidence that family planning is important for improving maternal health (by, among other things, reducing the number of maternal deaths simply by reducing the number of women who become pregnant). There is extensive evidence that family planning can be effectively provided through a community–based primary health care platform [30–32]. Had assessments of the effectiveness of community–based family planning been included in our review, we expect that the evidence for the effectiveness of CBPHC in improving maternal health would have been even more compelling.

Our inclusion of a wide variety of intervention packages precludes us from being able to make specific recommendations for or against intervention components in community–based approaches. However, other authors have summarized potential frameworks to select appropriate intervention package components [33,34]. The nature of intervention packages evolves with technology and with the emergence of new interventions. For example, mhealth strategies involving community health workers and women of reproductive age have the potential to link clients with services and promote utilization of services [35]. However, no studies assessing mHealth interventions were identified for our review. In addition, the lack of standardization of indicator measurement limits our ability to draw detailed conclusions. Finally, the richness of data set is such that only a limited analysis of the data is provided here. Further analyses are needed, as pointed out at several points in this paper.

## CONCLUSIONS

The evidence provided here supports the recommendation that CBPHC is an important component of a comprehensively–designed maternal health program – not only because of the direct effects it can have on reducing maternal morbidity and its potential to contribute to reductions in maternal mortality, but also because of its contributions to the promotion of appropriate facility utilization for ANC, childbirth, and referral of obstetrical emergencies. Finally, the closely related contributions that CBPHC can make to improving neonatal health are important as well but summarized in another article in this series [10].
