# Supplementary material for: Comprehensive review of the evidence regarding the effectiveness of community–based primary health care in improving maternal, neonatal and child health: 2. maternal health findings
Source: J Glob Health. 2017 Jun 29;7(1):010902. doi: 10.7189/jogh.07.010902 (PMC5491947; doi:10.7189/jogh.07.010902)
Supplement: Online Supplementary Document [file jogh-07-010902-s001.pdf]

Online Supplementary Document

Jennings et al. Comprehensive review of the evidence regarding the effectiveness of community–based primary health care in improving maternal, neonatal and child health: 2. maternal health findings

J Glob Health 2017;7:010902

Appendix S1

References for the Assessments Included in the Maternal Health Review

S1 AbdelRahman, S. H., Albashir, I. M., Hussein, S. A., Ahmed, M. E., Alfadil, S. M., & Mohamed, N. (2007). BDN programmes and the effect of medical students' interventions to promote child health in Sudan. East Mediterr Health J, 13(6), 1319-1329.

S2 Adam, M. B., Dillmann, M., Chen, M. K., Mbugua, S., Ndung'u, J., Mumbi, P., . . . Meissner, P. (2014). Improving maternal and newborn health: effectiveness of a community health worker program in rural Kenya. PLoS One, 9(8), e104027. doi:10.1371/journal.pone.0104027

S3 Adams, A. M., Nababan, H. Y., & Hanifi, S. M. (2015). Building social networks for maternal and newborn health in poor urban settlements: a cross-sectional study in Bangladesh. PLoS One, 10(4), e0123817. doi:10.1371/journal.pone.0123817

S4 Adventist Development Relief Agency/Cambodia. (2006). Child survival XVII - Final evaluation.

S5 Adventist Development Relief Agency/Guinea. (2005). Child survival XVI: Final Evaluation Siguiri Prefecture.

S6 Adventist Development Relief Agency/Nicaragua. (2006). Final Evaluation: Child Survival XVII.

S7 Afari, E. A., Nkrumah, F. K., Nakana, T., Sakatoku, H., Hori, H., & Binka, F. (1995). Impact of primary health care on child morbidiy and mortality. Central African Journal of Medicine, 41(5), 148-153.

S8 Aga Khan Foundation/Pakistan (2014). Chitral Child Survival Project, Chitral, Khyber Pakhtunkhwa, Pakistan: Final Evaluation.

S9 Agha, S. (2011). Impact of a maternal health voucher scheme on institutional delivery among low income women in Pakistan. Reprod Health, 8, 10. doi:10.1186/1742-4755-8-10

S10 Aghajanian, A., Mehryar, A. H., Ahmadnia, S., & Kazemipour, S. (2007). Impact of rural health development programme in the Islamic Republic of Iran on rural-urban disparities in health indicators. East Mediterr Health J, 13(6), 1466-1475.

S11 Ahluwalia, I. B., Robinson, D., Vallely, L., Gieseker, K. E., & Kabakama, A. (2010). Sustainability of community-capacity to promote safer motherhood in northwestern Tanzania: what remains? Glob Health Promot, 17(1), 39-49. doi:10.1177/1757975909356627

S12 Ahluwalia, I. B., Schmid, T., Kouletio, M., & Kanenda, O. (2003). An evaluation of a community-based approach to safe motherhood in northwestern Tanzania. Int J Gynaecol Obstet, 82(2), 231-240. doi:http://dx.doi.org/10.1016/s0020-7292(03)00081-x

S13 Ahmed, S., Ahmed, S., McKaig, C., Begum, N., Mungia, J., Norton, M., & Baqui, A. H. (2015). The Effect of Integrating Family Planning with a Maternal and Newborn Health Program on Postpartum Contraceptive Use and Optimal Birth Spacing in Rural Bangladesh. Stud Fam Plann, 46(3), 297-312. doi:10.1111/j.1728-4465.2015.00031.x

S14 Alehagen, S. A., Finnstrom, O., Hermansson, G. V., Somasundaram, K. V., Bangal, V. B., Patil, A., . . . Johansson, A. K. (2012). Nurse-based antenatal and child health care in rural India, implementation and effects - an Indian-Swedish collaboration. Rural Remote Health, 12, 2140.

S15 Alisjahbana, A., Williams, C., Dharmayanti, R., Hermawan, D., Kwast, B. E., & Koblinsky, M. (1995). An integrated village maternity service to improve referral patterns in a rural area in West-Java. Int J Gynaecol Obstet, 48 Suppl, S83-94. doi:http://dx.doi.org/10.1016/0020-7292(95)02323-5

S16 Alto, W. A., Albu, R. E., & Irabo, G. (1991). An alternative to unattended delivery--a training programme for village midwives in Papua New Guinea. Soc Sci Med, 32(5), 613-618. doi:http://dx.doi.org/10.1016/0277-9536(91)90296-o

S17 Alvarado, R., Zepeda, A., Rivero, S., Rico, N., Lopez, S., & Diaz, S. (1999). Integrated maternal and infant health care in the postpartum period in a poor neighborhood in Santiago, Chile. Stud Fam Plann, 30(2), 133-141. doi:http://dx.doi.org/10.1111/j.1728-4465.1999.00133.x

S18 Aracena, M., Krause, M., Perez, C., Mendez, M. J., Salvatierra, L., Soto, M., . . . Altimir, C. (2009). A cost-effectiveness evaluation of a home visit program for adolescent mothers. J Health Psychol, 14(7), 878-887. doi:10.1177/1359105309340988

S19 Asha/India (2008). Overview. Available: http://asha-india.org/

S21 Azad, K., Barnett, S., Banerjee, B., Shaha, S., Khan, K., Rego, A. R., . . . Costello, A. (2010). Effect of scaling up women's groups on birth outcomes in three rural districts in Bangladesh: a cluster-randomised controlled trial. Lancet, 375(9721), 1193-1202. doi:10.1016/S0140-6736(10)60142-0

S22 Babalola, S., Sakolsky, N., Vondrasek, C., Mounlom, D., Brown, J., & Tchupo, J. P. (2001). The impact of a community mobilization project on health-related knowledge and practices in Cameroon. J Community Health, 26(6), 459-477.

S23 Baqui, A. H., El-Arifeen, S., Darmstadt, G. L., Ahmed, S., Williams, E. K., Seraji, H. R., . . . Projahnmo Study, G. (2008). Effect of community-based newborn-care intervention package implemented through two service-delivery strategies in Sylhet district, Bangladesh: a cluster-randomised controlled trial. Lancet, 371(9628), 1936-1944. doi:10.1016/S0140-6736(08)60835-1

S24 Baqui, A. H., Rosecrans, A. M., Williams, E. K., Agrawal, P. K., Ahmed, S., Darmstadt, G. L., . . . Santosham, M. (2008). NGO facilitation of a government community-based maternal and neonatal health programme in rural India: improvements in equity. Health Policy Plan, 23(4), 234-243. doi:10.1093/heapol/czn012

S25 Baqui, A., Williams, E. K., Rosecrans, A. M., Agrawal, P. K., Ahmed, S., Darmstadt, G. L., . . . Santosham, M. (2008). Impact of an integrated nutrition and health programme on neonatal mortality in rural northern India. Bull World Health Organ, 86(10), 796-804, A. doi:http://dx.doi.org/10.2471/blt.07.042226

S26 Bashour, H. N., Kharouf, M. H., Abdulsalam, A. A., El Asmar, K., Tabbaa, M. A., & Cheikha, S. A. (2008). Effect of postnatal home visits on maternal/infant outcomes in Syria: a randomized controlled trial. Public Health Nurs, 25(2), 115-125. doi:10.1111/j.1525-1446.2008.00688.x
S27 Bhutta, Z. A., Memon, Z. A., Soofi, S., Salat, M. S., Cousens, S., & Martines, J. (2008). Implementing community-based perinatal care: results from a pilot study in rural Pakistan. Bull World Health Organ, 86(6), 452-459. doi:http://dx.doi.org/10.2471/blt.07.045849
S28 Bhutta, Z. A., Rizvi, A., Raza, F., Hotwani, S., Zaidi, S., Moazzam Hossain, S., . . . Bhutta, S. (2009). A comparative evaluation of multiple micronutrient and iron-folic acid supplementation during pregnancy in Pakistan: impact on pregnancy outcomes. Food Nutr Bull, 30(4 Suppl), S496-505. doi:http://dx.doi.org/10.1177/15648265090304s404
S29 Bhutta, Z. A., Soofi, S., Cousens, S., Mohammad, S., Memon, Z. A., Ali, I., . . . Martines, J. (2011). Improvement of perinatal and newborn care in rural Pakistan through community-based strategies: a cluster-randomised effectiveness trial. Lancet, 377(9763), 403-412. doi:10.1016/S0140-6736(10)62274-X
S30 Bryce, J., Gilroy, K., Jones, G., Hazel, E., Black, R. E., & Victora, C. G. (2008). The Retrospective Evaluation of ACSD: Cross-site analyses and conclusions.
S31 Bryce J, Gilroy K, Jones G, Hazel E, Black RE, Victora CG.(2010). The Accelerated Child Survival and Development programme in west Africa: a retrospective evaluation. 2010;375:572-82. Doi: S0140-6736(09)62060-2 [pii] 10.1016/S0140-6736(09)62060-2
S32 Callaghan-Koru, J. A., Nonyane, B. A., Guenther, T., Sitrin, D., Ligowe, R., Chimbalanga, E., . . . Baqui, A. H. (2013). Contribution of community-based newborn health promotion to reducing inequities in healthy newborn care practices and knowledge: evidence of improvement from a three-district pilot program in Malawi. BMC Public Health, 13, 1052. doi:10.1186/1471-2458-13-1052
S33 Care /Nicaragua (2008). Child Survival Project (CSP) XVIII: Phase two - Final Evaluation.
S34 Care/Mozambique. (2006). Child survial project: Final evaluation.
S35 Care/Sierra Leone (2008). Child survival project 'For Di Pikin Dem Wel Bodi', Koinadugu District, Northern Region, Sierra Leone: Final evaluation.
S36 CB-MNC Nepal (2007). Community-based maternal and neonatal care program (CB-MNC): Summative report on program activities and results in Banka, Jhapa and Kanchanpur districts from September 2005 - September 2007.
S37 Cesar, J. A., Mendoza-Sassi, R. A., Ulmi, E. F., Dall'Agnol, M. M., & Neumann, N. A. (2008). [Effects of different home visit strategies on prenatal care in Southern Brazil]. Cad Saude Publica, 24(11), 2614-2622. doi:http://dx.doi.org/10.1590/s0102-311x2008001100016
S38 Chen, L. C., Rahman, M., D'Souza, S., Chakraborty, J., Sardar, A. M., & Yunus, M. (1983). Mortality impact of an MCH-FP program in Matlab, Bangladesh. Stud Fam Plann, 14(8-9), 199-209. doi:http://dx.doi.org/10.2307/1966412
S39 Colbourn, T., Nambiar, B., Bondo, A., Makwenda, C., Tsetekani, E., Makonda-Ridley, A., . . . Costello, A. (2013). Effects of quality improvement in health facilities and community mobilization through women's groups on maternal, neonatal and perinatal mortality in three districts of Malawi: MaiKhanda, a cluster randomized controlled effectiveness trial. Int Health, 5(3), 180-195. doi:10.1093/inthealth/iht011
S40 Curamericas Global/Liberia (2013). Final evaluation for Nehnwaa child survival project: Census-Based Impact-Oriented methodology for community-based primary health care in Nimba country, Liberia.

S41 Curamericas/Bolivia. (2007). Providing child survival services to rural and peri-urban populations in Bolivia - Final Evaluation Report.
S42 Curamericas/Guatemala (2007). Census-Based, Impact-Oriented Child Survival Project. October 1, 2002 - September 30, 2007: Final Evaluation Report
S43 Curamericas/Guatemala (2015). Community-Based, Impact-Oriented Child Survial in Huehuetenango, Guatemala: Focused strategic assessment.

S44 Darmstadt, G. L., Choi, Y., Arifeen, S. E., Bari, S., Rahman, S. M., Mannan, I., . . . Bangladesh Projahnmo-2 Mirzapur Study, G. (2010). Evaluation of a cluster-randomized controlled trial of a package of community-based maternal and newborn interventions in Mirzapur, Bangladesh. PLoS One, 5(3), e9696. doi:10.1371/journal.pone.0009696
S45 Derman, R. J., Kodkany, B. S., Goudar, S. S., Geller, S. E., Naik, V. A., Bellad, M. B., . . . Moss, N. (2006). Oral misoprostol in preventing postpartum haemorrhage in resource-poor communities: a randomised controlled trial. Lancet, 368(9543), 1248-1253. doi:10.1016/S0140-6736(06)69522-6
S46 Dutt, D., & Srinivasa, D. K. (1997). Impact of maternal and child health strategy on child survival in a rural community of Pondicherry. Indian Pediatr, 34(9), 785-792.
S47 Emond, A., Pollock, J., Da Costa, N., Maranhao, T., & Macedo, A. (2002). The effectiveness of community-based interventions to improve maternal and infant health in the Northeast of Brazil. Rev Panam Salud Publica, 12(2), 101-110. doi:http://dx.doi.org/10.1590/s1020-49892002000800005
S48 Ezeanolue, E. E., Obiefune, M. C., Ezeanolue, C. O., Ehiri, J. E., Osuji, A., Ogidi, A. G., . . . Ogedegbe, G. (2015). Effect of a congregation-based intervention on uptake of HIV testing and linkage to care in pregnant women in Nigeria (Baby Shower): a cluster randomised trial. Lancet Glob Health, 3(11), e692-700. doi:10.1016/S2214-109X(15)00195-3
S49 Fathima, F. N., Raju, M., Varadharajan, K. S., Krishnamurthy, A., Ananthkumar, S. R., & Mony, P. K. (2015). Assessment of 'accredited social health activists'-a national community health volunteer scheme in Karnataka State, India. J Health Popul Nutr, 33(1), 137-145.
S50 Fauveau, V., Wojtyniak, B., Chakraborty, J., Sarder, A. M., & Briend, A. (1990). The effect of maternal and child health and family planning services on mortality: is prevention enough? BMJ, 301(6743), 103-107. doi:http://dx.doi.org/10.1136/bmj.301.6743.103
S51 Fauveau, V., Stewart, K., Khan, S. A., & Chakraborty, J. (1991). Effect on mortality of community-based maternity-care programme in rural Bangladesh. Lancet, 338(8776), 1183-1186. doi:http://dx.doi.org/10.1016/0140-6736(91)92041-y
S52 Findley, S. E., Uwemedimo, O. T., Doctor, H. V., Green, C., Adamu, F., & Afenyadu, G. Y. (2013). Comparison of high- versus low-intensity community health worker intervention to promote newborn and child health in Northern Nigeria. Int J Womens Health, 5, 717-728. doi:10.2147/IJWH.S49785
S53 Findley, S. E., Doctor, H. V., Ashir, G. M., Kana, M. A., Mani, A. S., Green, C., & Afenyadu, G. Y. (2015). Reinvigorating health systems and community-based services to improve maternal health outcomes: case study from northern Nigeria. J Prim Care Community Health, 6(2), 88-99. doi:10.1177/2150131914549383
S54 Foord, F. (1995). Gambia: evaluation of the mobile health care service in West Kiang district. World Health Stat Q, 48(1), 18-22.

S55 Fox-Rushby, J. A., & Foord, F. (1996). Costs, effects and cost-effectiveness analysis of a mobile maternal health care service in West Kiang, The Gambia. Health Policy, 35(2), 123-143. doi:http://dx.doi.org/10.1016/0168-8510(95)00774-1

S56 Future Generations/Afghanistan (2006). Community health worker training for women's empowerment in Afghanistan: Summary report.

S57 Future Generations/Peru (2007). CLAS Las Moras: Intermediate Evaluation.

S58 Gloyd, S., Floriano, F., Seunda, M., Chadreque, M. A., Nyangezi, J. M., & Platas, A. (2001). Impact of traditional birth attendant training in Mozambique: a controlled study. J Midwifery Womens Health, 46(4), 210-216. doi:http://dx.doi.org/10.1016/s1526-9523(01)00142-8

S59 Haver, J., Brieger, W., Zoungrana, J., Ansari, N., & Kagoma, J. (2015). Experiences engaging community health workers to provide maternal and newborn health services: implementation of four programs. Int J Gynaecol Obstet, 130 Suppl 2, S32-39. doi:10.1016/j.ijgo.2015.03.006

S60 Helen Keller International (2003). Community-based Iron+Folic Acid Supplementation and Nutrition Education for Pregnant Women: Manica Province. Maputo, Mozambique

S61 Hodgins, S., McPherson, R., Suvedi, B. K., Shrestha, R. B., Silwal, R. C., Ban, B., . . . Baqui, A. H. (2010). Testing a scalable community-based approach to improve maternal and neonatal health in rural Nepal. J Perinatol, 30(6), 388-395. doi:10.1038/jp.2009.181

S62 Ibrahim, S. A., Omer, M. I., Amin, I. K., Babiker, A. G., & Rushwan, H. (1992). The role of the village midwife in detection of high risk pregnancies and newborns. Int J Gynaecol Obstet, 39(2), 117-122. doi:http://dx.doi.org/10.1016/0020-7292(92)90907-z

S63 Jokhio, A. H., Winter, H. R., & Cheng, K. K. (2005). An intervention involving traditional birth attendants and perinatal and maternal mortality in Pakistan. N Engl J Med, 352(20), 2091-2099. doi:10.1056/NEJMsa042830

S64 Kandeh, H. B., Leigh, B., Kanu, M. S., Kuteh, M., Bangura, J., & Seisay, A. L. (1997). Community motivators promote use of emergency obstetric services in rural Sierra Leone. The Freetown/Makeni PMM Team. Int J Gynaecol Obstet, 59 Suppl 2, S209-218. doi:http://dx.doi.org/10.1016/s0020-7292(97)00167-7

S65 Kapungu, C. T., Mensah-Homiah, J., Akosah, E., Asare, G., Carnahan, L., Frimpong, M. A., . . . Ghana, P. P. H. S. G. (2013). A community-based continuum of care model for the prevention of postpartum hemorrhage in rural Ghana. Int J Gynaecol Obstet, 120(2), 156-159. doi:10.1016/j.ijgo.2012.08.021

S66 Karim, A. M., Admassu, K., Schellenberg, J., Alemu, H., Getachew, N., Ameha, A., . . . Betemariam, W. (2013). Effect of ethiopia's health extension program on maternal and newborn health care practices in 101 rural districts: a dose-response study. PLoS One, 8(6), e65160. doi:10.1371/journal.pone.0065160

S67 Kema, K. M., Komwihangiro, J., & Kimaro, S. (2012). Integrated community based child survival, reproductive health and water and sanitation program in Mkuranga district, Tanzania: a replicable model of good practices in community based health care. Pan Afr Med J, 13 Suppl 1, 11.

S68 Khan, M. A., & Ahmed, S. M. (2009). The "Birthing Hut" facilities of MANOSHI: A Two-Part Paper, Exploring the Inception and Post-Inception Phases of Urban Delivery Centres of Dhaka.

S69 Kim, M. H., Ahmed, S., Buck, W. C., Preidis, G. A., Hosseinipour, M. C., Bhalakia, A., . . . Kline, M. W. (2012). The Tingathe programme: a pilot intervention using community

health workers to create a continuum of care in the prevention of mother to child transmission of HIV (PMTCT) cascade of services in Malawi. J Int AIDS Soc, 15 Suppl 2, 17389. doi:10.7448/IAS.15.4.17389
S70 Koenig, M. A., Fauveau, V., Chowdhury, A. I., Chakraborty, J., & Khan, M. A. (1988). Maternal mortality in Matlab, Bangladesh: 1976-85. Stud Fam Plann, 19(2), 69-80. doi:http://dx.doi.org/10.2307/1966492
S71 Kohli, C., Kishore, J., Sharma, S., & Nayak, H. (2015). Knowledge and practice of Accredited Social Health Activists for maternal healthcare delivery in Delhi. J Family Med Prim Care, 4(3), 359-363. doi:10.4103/2249-4863.161317
S72 Kumar, V., Kumar, A., Das, V., Srivastava, N. M., Baqui, A. H., Santosham, M., . . . Saksham Study, G. (2012). Community-driven impact of a newborn-focused behavioral intervention on maternal health in Shivgarh, India. Int J Gynaecol Obstet, 117(1), 48-55. doi:10.1016/j.ijgo.2011.10.031
S73 Lamb, W. H., Foord, F. A., Lamb, C. M., & Whitehead, R. G. (1984). Changes in maternal and child mortality rates in three isolated Gambian villages over ten years. Lancet, 2(8408), 912-914. doi:http://dx.doi.org/10.1016/s0140-6736(84)90664-0
S74 Langston, A., Weiss, J., Landegger, J., Pullum, T., Morrow, M., Kabadege, M., . . . Sarriot, E. (2014). Plausible role for CHW peer support groups in increasing care-seeking in an integrated community case management project in Rwanda: a mixed methods evaluation. Glob Health Sci Pract, 2(3), 342-354. doi:10.9745/GHSP-D-14-00067
S75 Larocque, R., Casapia, M., Gotuzzo, E., MacLean, J. D., Soto, J. C., Rahme, E., & Gyorkos, T. W. (2006). A double-blind randomized controlled trial of antenatal mebendazole to reduce low birthweight in a hookworm-endemic area of Peru. Trop Med Int Health, 11(10), 1485-1495. doi:10.1111/j.1365-3156.2006.01706.x
S76 Levy-Bruhl, D., Soucat, A., Osseni, R., Ndiaye, J. M., Dieng, B., De Bethune, X., . . . Knippenberg, R. (1997). The Bamako Initiative in Benin and Guinea: improving the effectiveness of primary health care. Int J Health Plann Manage, 12 Suppl 1, S49-79. doi:10.1002/(SICI)1099-1751(199706)12:1+<S49::AID-HPM466>3.0.CO;2-P
S77 Lim, S. S., Dandona, L., Hoisington, J. A., James, S. L., Hogan, M. C., & Gakidou, E. (2010). India's Janani Suraksha Yojana, a conditional cash transfer programme to increase births in health facilities: an impact evaluation. Lancet, 375(9730), 2009-2023. doi:10.1016/S0140-6736(10)60744-1
S78 Lori, J. R., Amable, E. E., Mertz, S. G., & Moriarty, K. (2012). Behavior change following implementation of home-based life-saving skills in Liberia, West Africa. J Midwifery Womens Health, 57(5), 495-501. doi:10.1111/j.1542-2011.2012.00172.x
S79 Lori, J. R., Munro, M. L., Rominski, S., Williams, G., Dahn, B. T., Boyd, C. J., . . . Gwenegale, W. (2013). Maternity waiting homes and traditional midwives in rural Liberia. Int J Gynaecol Obstet, 123(2), 114-118. doi:10.1016/j.ijgo.2013.05.024
S80 Lunsford, S. S., Fatta, K., Stover, K. E., & Shrestha, R. (2015). Supporting close-to-community providers through a community health system approach: case examples from Ethiopia and Tanzania. Hum Resour Health, 13, 12. doi:10.1186/s12960-015-0006-6
S81 Magnani, R. J. (1995). Strengthening MCH/FP at the grassroots level: People's Republic of China. United Nations Population Fund.
S82 Management Sciences for Health/REACH (2006). Rural expansion of Afganistan's community-based healthcare program: Measuring program outcomes through household surveys.

S83 Manandhar, D. S., Osrin, D., Shrestha, B. P., Mesko, N., Morrison, J., Tumbahangphe, K. M., . . . Members of the MIRA Mawankpur Study Team (2004). Effect of a participatory intervention with women's groups on birth outcomes in Nepal: cluster-randomised controlled trial. Lancet, 364(9438), 970-979. doi:10.1016/S0140-6736(04)17021-9
S84 Martin, K. (2008). Overview of Asha/India
S85 Mathur, H. N., Damodar, Sharma, P. N., & Jain, T. P. (1979). The impact of training traditional birth attendants on the utilisation of maternal health services. J Epidemiol Community Health, 33(2), 142-144. doi:http://dx.doi.org/10.1136/jech.33.2.142
S86 Mbonye, A. K., Bygbjerg, I., & Magnussen, P. (2007). Intermittent preventive treatment of malaria in pregnancy: evaluation of a new delivery approach and the policy implications for malaria control in Uganda. Health Policy, 81(2-3), 228-241. doi:10.1016/j.healthpol.2006.05.018
S87 Mbonye, A. K., Bygbjerg, I. C., & Magnussen, P. (2008). Intermittent preventive treatment of malaria in pregnancy: a new delivery system and its effect on maternal health and pregnancy outcomes in Uganda. Bull World Health Organ, 86(2), 93-100. doi:http://dx.doi.org/10.2471/blt.07.041822
S88 Mbonye, A. K., Bygbjerg, I., & Magnussen, P. (2008). Intermittent preventive treatment of malaria in pregnancy: a community-based delivery system and its effect on parasitemia, anemia and low birth weight in Uganda. Int J Infect Dis, 12(1), 22-29. doi:10.1016/j.ijid.2006.10.008
S89 Mbonye, A. K., Hansen, K. S., Bygbjerg, I. C., & Magnussen, P. (2008). Intermittent preventive treatment of malaria in pregnancy: the incremental cost-effectiveness of a new delivery system in Uganda. Trans R Soc Trop Med Hyg, 102(7), 685-693. doi:10.1016/j.trstmh.2008.04.016
S90 Mbonye, A. K., Schultz Hansen, K., Bygbjerg, I. C., & Magnussen, P. (2008). Effect of a community-based delivery of intermittent preventive treatment of malaria in pregnancy on treatment seeking for malaria at health units in Uganda. Public Health, 122(5), 516-525. doi:10.1016/j.puhe.2007.07.024
S91 McPherson, R. A., Khadka, N., Moore, J. M., & Sharma, M. (2006). Are birth-preparedness programmes effective? Results from a field trial in Siraha district, Nepal. J Health Popul Nutr, 24(4), 479-488.
S92 Memon, Z. A., Khan, G. N., Soofi, S. B., Baig, I. Y., & Bhutta, Z. A. (2015). Impact of a community-based perinatal and newborn preventive care package on perinatal and neonatal mortality in a remote mountainous district in Northern Pakistan. BMC Pregnancy Childbirth, 15, 106. doi:10.1186/s12884-015-0538-8
S93 Mercer, A., Khan, M. H., Daulatuzzaman, M., & Reid, J. (2004). Effectiveness of an NGO primary health care programme in rural Bangladesh: evidence from the management information system. Health Policy Plan, 19(4), 187-198. doi:http://dx.doi.org/10.1093/heapol/czh024
S94 Midhet, F., & Becker, S. (2010). Impact of community-based interventions on maternal and neonatal health indicators: Results from a community randomized trial in rural Balochistan, Pakistan. Reprod Health, 7, 30. doi:10.1186/1742-4755-7-30
S95 Mobeen, N., Durocher, J., Zuberi, N., Jahan, N., Blum, J., Wasim, S., . . . Hatcher, J. (2011). Administration of misoprostol by trained traditional birth attendants to prevent postpartum haemorrhage in homebirths in Pakistan: a randomised placebo-controlled trial. BJOG, 118(3), 353-361. doi:10.1111/j.1471-0528.2010.02807.x

S96 Mohan, D., Gupta, S., LeFevre, A., Bazant, E., Killewo, J., & Baqui, A. H. (2015). Determinants of postnatal care use at health facilities in rural Tanzania: multilevel analysis of a household survey. BMC Pregnancy Childbirth, 15, 282. doi:10.1186/s12884-015-0717-7

S97 More, N. S., Bapat, U., Das, S., Alcock, G., Patil, S., Porel, M., . . . Osrin, D. (2012). Community mobilization in Mumbai slums to improve perinatal care and outcomes: a cluster randomized controlled trial. PLoS Med, 9(7), e1001257. doi:10.1371/journal.pmed.1001257
S98 Morris, S. S., Flores, R., Olinto, P., & Medina, J. M. (2004). Monetary incentives in primary health care and effects on use and coverage of preventive health care interventions in rural Honduras: cluster randomised trial. Lancet, 364(9450), 2030-2037. doi:10.1016/S0140-6736(04)17515-6
S99 Morrison, J., Tamang, S., Mesko, N., Osrin, D., Shrestha, B., Manandhar, M., . . . Costello, A. (2005). Women's health groups to improve perinatal care in rural Nepal. BMC Pregnancy Childbirth, 5(1), 6. doi:10.1186/1471-2393-5-6
S100 Msyamboza, K. P., Savage, E. J., Kazembe, P. N., Gies, S., Kalanda, G., D'Alessandro, U., & Brabin, B. J. (2009). Community-based distribution of sulfadoxine-pyrimethamine for intermittent preventive treatment of malaria during pregnancy improved coverage but reduced antenatal attendance in southern Malawi. Trop Med Int Health, 14(2), 183-189. doi:10.1111/j.1365-3156.2008.02197.x
S101 Mullany, L. C., Lee, T. J., Yone, L., Lee, C. I., Teela, K. C., Paw, P., . . . Beyrer, C. (2010). Impact of community-based maternal health workers on coverage of essential maternal health interventions among internally displaced communities in eastern Burma: the MOM project. PLoS Med, 7(8), e1000317. doi:10.1371/journal.pmed.1000317
S102 Mushamiri, I., Luo, C., Iiams-Hauser, C., & Ben Amor, Y. (2015). Evaluation of the impact of a mobile health system on adherence to antenatal and postnatal care and prevention of mother-to-child transmission of HIV programs in Kenya. BMC Public Health, 15, 102. doi:10.1186/s12889-015-1358-5
S103 Mushi, D., Mpembeni, R., & Jahn, A. (2010). Effectiveness of community based Safe Motherhood promoters in improving the utilization of obstetric care. The case of Mtwara Rural District in Tanzania. BMC Pregnancy Childbirth, 10, 14. doi:10.1186/1471-2393-10-14
S104 Nahar, T., Azad, K., Aumon, B. H., Younes, L., Shaha, S., Kuddus, A., . . . Fottrell, E. (2012). Scaling up community mobilisation through women's groups for maternal and neonatal health: experiences from rural Bangladesh. BMC Pregnancy Childbirth, 12, 5. doi:10.1186/1471-2393-12-5
S105 Ndiaye, M., Siekmans, K., Haddad, S., & Receveur, O. (2009). Impact of a positive deviance approach to improve the effectiveness of an iron-supplementation program to control nutritional anemia among rural Senegalese pregnant women. Food Nutr Bull, 30(2), 128-136. doi:http://dx.doi.org/10.1177/156482650903000204
S106 Newlands, D., Yugbare-Belemsaga, D., Ternent, L., Hounton, S., & Chapman, G. (2008). Assessing the costs and cost-effectiveness of a skilled care initiative in rural Burkina Faso. Trop Med Int Health, 13 Suppl 1, 61-67. doi:10.1111/j.1365-3156.2008.02088.x
S107 Ngabo, F., Nguimfack, J., Nwaigwe, F., Mugeni, C., Muhoza, D., Wilson, D. R., . . . Binagwaho, A. (2012). Designing and Implementing an Innovative SMS-based alert system (RapidSMS-MCH) to monitor pregnancy and reduce maternal and child deaths in Rwanda. Pan Afr Med J, 13, 31.
S108 Nonyane, B. A., Kc, A., Callaghan-Koru, J. A., Guenther, T., Sitrin, D., Syed, U., . . . Baqui, A. H. (2015). Equity improvements in maternal and newborn care indicators: results from the Bardiya district of Nepal. Health Policy Plan. doi:10.1093/heapol/czv077

S109 Okeibunor, J. C., Orji, B. C., Brieger, W., Ishola, G., Otolorin, E., Rawlins, B., . . . Fink, G. (2011). Preventing malaria in pregnancy through community-directed interventions: evidence from Akwa Ibom State, Nigeria. Malar J, 10, 227. doi:10.1186/1475-2875-10-227
S110 Omer, K., Mhatre, S., Ansari, N., Laucirica, J., & Andersson, N. (2008). Evidence-based training of frontline health workers for door-to-door health promotion: a pilot randomized controlled cluster trial with Lady Health Workers in Sindh Province, Pakistan. Patient Educ Couns, 72(2), 178-185. doi:10.1016/j.pec.2008.02.018
S111 Opoku, S. A., Kyei-Faried, S., Twum, S., Djan, J. O., Browne, E. N., & Bonney, J. (1997). Community education to improve utilization of emergency obstetric services in Ghana. The Kumasi PMM Team. Int J Gynaecol Obstet, 59 Suppl 2, S201-207. doi:http://dx.doi.org/10.1016/s0020-7292(97)00166-5
S112 O'Rourke, K., Howard-Grabman, L., & Seoane, G. (1998). Impact of community organization of women on perinatal outcomes in rural Bolivia. Rev Panam Salud Publica, 3(1), 9-14. doi:http://dx.doi.org/10.1590/s1020-49891998000100002
S113 Owusu-Agyei, S., Awini, E., Anto, F., Mensah-Afful, T., Adjuik, M., Hodgson, A., . . . Binka, F. (2007). Assessing malaria control in the Kassena-Nankana district of northern Ghana through repeated surveys using the RBM tools. Malar J, 6, 103. doi:10.1186/1475-2875-6-103
S114 Perez, F., Mukotekwa, T., Miller, A., Orne-Gliemann, J., Glenshaw, M., Chitsike, I., & Dabis, F. (2004). Implementing a rural programme of prevention of mother-to-child transmission of HIV in Zimbabwe: first 18 months of experience. Trop Med Int Health, 9(7), 774-783. doi:10.1111/j.1365-3156.2004.01264.x
S115 Plan/Nepal (2006). For our mothers and children: Scaling success to the district level Plan Nepal Child Survival XVIII Cost Extension.
S116 Prata, N., Ejembi, C., Fraser, A., Shittu, O., & Minkler, M. (2012). Community mobilization to reduce postpartum hemorrhage in home births in northern Nigeria. Soc Sci Med, 74(8), 1288-1296. doi:10.1016/j.socscimed.2011.11.035
S117 Purdin, S., Khan, T., & Saucier, R. (2009). Reducing maternal mortality among Afghan refugees in Pakistan. Int J Gynaecol Obstet, 105(1), 82-85. doi:10.1016/j.ijgo.2008.12.021
S118 Rahman, A., Malik, A., Sikander, S., Roberts, C., & Creed, F. (2008). Cognitive behaviour therapy-based intervention by community health workers for mothers with depression and their infants in rural Pakistan: a cluster-randomised controlled trial. Lancet, 372(9642), 902-909. doi:10.1016/S0140-6736(08)61400-2
S119 Rahman, M., Jhohura, F. T., Mistry, S. K., Chowdhury, T. R., Ishaque, T., Shah, R., & Afsana, K. (2015). Assessing Community Based Improved Maternal Neonatal Child Survival (IMNCS) Program in Rural Bangladesh. PLoS One, 10(9), e0136898. doi:10.1371/journal.pone.0136898
S120 Rajbhandari, S., Hodgins, S., Sanghvi, H., McPherson, R., Pradhan, Y. V., Baqui, A. H., & Misoprostol Study, G. (2010). Expanding uterotonic protection following childbirth through community-based distribution of misoprostol: operations research study in Nepal. Int J Gynaecol Obstet, 108(3), 282-288. doi:10.1016/j.ijgo.2009.11.006
S121 Rath, S., Nair, N., Tripathy, P. K., Barnett, S., Rath, S., Mahapatra, R., . . . Prost, A. (2010). Explaining the impact of a women's group led community mobilisation intervention on maternal and newborn health outcomes: the Ekjut trial process evaluation. BMC Int Health Hum Rights, 10, 25. doi:10.1186/1472-698X-10-25

S122 Rotheram-Borus, M. J., Tomlinson, M., le Roux, I. M., Harwood, J. M., Comulada, S., O'Connor, M. J., . . . Worthman, C. M. (2014). A cluster randomised controlled effectiveness trial evaluating perinatal home visiting among South African mothers/infants. PLoS One, 9(10), e105934. doi:10.1371/journal.pone.0105934
S123 Sanghvi, H., Ansari, N., Prata, N. J., Gibson, H., Ehsan, A. T., & Smith, J. M. (2010). Prevention of postpartum hemorrhage at home birth in Afghanistan. Int J Gynaecol Obstet, 108(3), 276-281. doi:10.1016/j.ijgo.2009.12.003
S124 SANRU III (2006). Final Evaluation.
S125 Save the Children/Ethiopia (2006). Essential Services for Maternal and Child Survival in Ethiopia: Mobilizing the Traditional and Public Health Sectors and Informing Programming for Pastoralist Populations.
S126 Save the Children/Guinea (2006). Child Survival 18-Guinea final evaluation report: Community health Initiative for the districts of Kouroussa and Mandiana Guinea.
S127 Save the Children/Zambia (2014). Lufwanyama Integrated Neonatal and Child Health Projec in Zambia (LINCHPIN): Final Evaluation.
S128 Seim, A. R., Alassoum, Z., Bronzan, R. N., Mainassara, A. A., Jacobsen, J. L., & Gali, Y. A. (2014). Pilot community-mobilization program reduces maternal and perinatal mortality and prevents obstetric fistula in Niger. Int J Gynaecol Obstet, 127(3), 269-274. doi:10.1016/j.ijgo.2014.06.016
S129 Shah, P. M., Selwyn, B. J., Shah, K., & Kumar, V. (1993). Evaluation of the home-based maternal record: a WHO collaborative study. Bull World Health Organ, 71(5), 535-548.
S130 Shamsuddin, L., Nahar, K., Nasrin, B., Nahar, S., Tamanna, S., Kabir, R. M., . . . Anwary, S. A. (2005). Use of parenteral magnesium sulphate in eclampsia and severe pre-eclampsia cases in a rural set up of Bangladesh. Bangladesh Med Res Counc Bull, 31(2), 75-82.
S131 Shetty, A. K., Mhazo, M., Moyo, S., von Lieven, A., Mateta, P., Katzenstein, D. A., . . . Bassett, M. T. (2005). The feasibility of voluntary counselling and HIV testing for pregnant women using community volunteers in Zimbabwe. Int J STD AIDS, 16(11), 755-759. doi:10.1258/095646205774763090
S132 Shrestha, J. R., Manandhar, D. S., Manandhar, S. R., Adhikari, D., Rai, C., Rana, H., . . . Pradhan, A. (2015). Maternal and Neonatal Health Knowledge, Service Quality and Utilization: Findings from a Community Based Quasi-experimental Trial in Arghakhanchi District of Nepal. J Nepal Health Res Counc, 13(29), 78-83.
S133 Sibley, L., Buffington, S. T., & Haileyesus, D. (2004). The American College of Nurse-Midwives' home-based lifesaving skills program: a review of the Ethiopia field test. J Midwifery Womens Health, 49(4), 320-328. doi:10.1016/j.jmwh.2004.03.013
S134 Skinner, J., & Rathavy, T. (2009). Design and evaluation of a community participatory, birth preparedness project in Cambodia. Midwifery, 25(6), 738-743. doi:10.1016/j.midw.2008.01.006
S135 Smith, A., Sabido, M., Camey, E., Batres, A., & Casabona, J. (2015). Lessons learned from integrating simultaneous triple point-of-care screening for syphilis, hepatitis B, and HIV in prenatal services through rural outreach teams in Guatemala. Int J Gynaecol Obstet, 130 Suppl 1, S70-72. doi:10.1016/j.ijgo.2015.04.009
S136 Smith, J. M., Baawo, S. D., Subah, M., Sirtor-Gbassie, V., Howe, C. J., Ishola, G., . . . Dwivedi, V. (2014). Advance distribution of misoprostol for prevention of postpartum hemorrhage (PPH) at home births in two districts of Liberia. BMC Pregnancy Childbirth, 14, 189. doi:10.1186/1471-2393-14-189

S137 Smith, J. M., Dimiti, A., Dwivedi, V., Ochieng, I., Dalaka, M., Currie, S., . . . McKaig, C. (2014). Advance distribution of misoprostol for the prevention of postpartum hemorrhage in South Sudan. Int J Gynaecol Obstet, 127(2), 183-188. doi:10.1016/j.ijgo.2014.05.016
S138 Stanton, C. K., Newton, S., Mullany, L. C., Cofie, P., Tawiah Agyemang, C., Adiibokah, E., . . . Owusu-Agyei, S. (2013). Effect on postpartum hemorrhage of prophylactic oxytocin (10 IU) by injection by community health officers in Ghana: a community-based, cluster-randomized trial. PLoS Med, 10(10), e1001524. doi:10.1371/journal.pmed.1001524
S139 Syed, U., Asiruddin, S., Helal, M. S., Mannan, II, & Murray, J. (2006). Immediate and early postnatal care for mothers and newborns in rural Bangladesh. J Health Popul Nutr, 24(4), 508-518.
S140 Thiam, L., & World Vision/Senegal (1994, Oct 2-7, 1994). Impact of the Thies CSP on the Health Knowledge and Practices of Mothers Living in the Sub-district of Niakhene (Thies region). Paper presented at the Community Impact of PVO Child Survival Efforts: 1995 - 1994, Bangalore, Karnataka, India.
S141 Timsa, L., Marrone, G., Ekirapa, E., & Waiswa, P. (2015). Strategies for helping families prepare for birth: experiences from eastern central Uganda. Glob Health Action, 8, 23969. doi:10.3402/gha.v8.23969
S142 Tripathy, P., Nair, N., Barnett, S., Mahapatra, R., Borghi, J., Rath, S., . . . Costello, A. (2010). Effect of a participatory intervention with women's groups on birth outcomes and maternal depression in Jharkhand and Orissa, India: a cluster-randomised controlled trial. Lancet, 375(9721), 1182-1192. doi:10.1016/S0140-6736(09)62042-0
S143 Turan, J. M., & Say, L. (2003). Community-based antenatal education in Istanbul, Turkey: effects on health behaviours. Health Policy Plan, 18(4), 391-398. doi:http://dx.doi.org/10.1093/heapol/czg047
S144 Turan, J. M., Tesfagiorghis, M., & Polan, M. L. (2011). Evaluation of a community intervention for promotion of safe motherhood in Eritrea. J Midwifery Womens Health, 56(1), 8-17. doi:10.1111/j.1542-2011.2010.00001.x
S145 USAID/Burundi (2013). Community health systems strengthening in Cibitoke province, Burundi: Mabayi child survival project final evaluation report.
S146 Uzondu, C. A., Doctor, H. V., Findley, S. E., Afenyadu, G. Y., & Ager, A. (2015). Female health workers at the doorstep: a pilot of community-based maternal, newborn, and child health service delivery in northern Nigeria. Glob Health Sci Pract, 3(1), 97-108. doi:10.9745/GHSP-D-14-00117
S147 Vision Eritera & the Swiss Red Cross (2007). Community-based health care project, Eritrea (2003-2007): Final Evaluation Report.
S148 Waiswa, P., Pariyo, G., Kallander, K., Akuze, J., Namazzi, G., Ekirapa-Kiracho, E., . . . Uganda Newborn Study, T. (2015). Effect of the Uganda Newborn Study on care-seeking and care practices: a cluster-randomised controlled trial. Glob Health Action, 8, 24584. doi:10.3402/gha.v8.24584
S149 Wajid, A., White, F., & Karim, M. S. (2013). Community health workers and health care delivery: evaluation of a women's reproductive health care project in a developing country. PLoS One, 8(9), e75476. doi:10.1371/journal.pone.0075476
S150 Wangalwa, G., Cudjoe, B., Wamalwa, D., Machira, Y., Ofware, P., Ndirangu, M., & Ilako, F. (2012). Effectiveness of Kenya's Community Health Strategy in delivering community-based maternal and newborn health care in Busia County, Kenya: non-randomized pre-test post test study. Pan Afr Med J, 13 Suppl 1, 12.

S151 Williamson, N. E., Parado, J. P., & Maturan, E. G. (1983). Providing maternal and child health-family planning services to a large rural population: results of the Bohol Project, Philippines. Am J Public Health, 73(1), 62-71. doi:http://dx.doi.org/10.2105/ajph.73.1.62
S152 Zhenxuan, X. (1995). China: Lowering maternal mortality in Miyun county, Beijing. World Health Stat Q, 48(1), 11-14.
